# Clinical Characteristics, Co-Mutations, and Treatment Outcomes in Advanced Non-Small-Cell Lung Cancer Patients With the BRAF-V600E Mutation

**DOI:** 10.3389/fonc.2022.911303

**Published:** 2022-06-22

**Authors:** Jingjing Qu, Qian Shen, Yuping Li, Farhin Shaheed Kalyani, Li Liu, Jianya Zhou, Jianying Zhou

**Affiliations:** ^1^ Department of Respiratory Disease, Thoracic Disease Center, The First Affiliated Hospital, College of Medicine, Zhejiang University, Hangzhou, China; ^2^ The Clinical Research Center for Respiratory Diseases of Zhejiang Province, Hangzhou, China; ^3^ Department of Respiratory Disease, The First Affiliated Hospital of Wenzhou Medical University, Wenzhou, China; ^4^ Lung Cancer and Gastroenterology Department, Hunan Cancer Hospital, Affiliated Tumor Hospital of Xiangya Medical School of Central South University, Changsha, China

**Keywords:** non-small cell lung cancer, BRAF-V600E, dabrafenib plus trametinib, co-mutations, treatment outcomes

## Abstract

**Background:**

Limited treatment outcome data is available for advanced non-small cell lung cancer (NSCLC) patients with BRAF V600E mutations. In this multicenter study, we describe therapeutic options and survival outcomes for patients with mutated BRAF V600E.

**Method:**

This was a retrospective study in which BRAF V600E-mutated advanced NSCLC patients were retrospectively recruited between January 2015 and December 2021 and had their clinical characteristics, co-mutations, and treatment efficacy assessed.

**Results:**

Fifty-three patients with BRAF V600E-mutant advanced NSCLC were included in the study, of which 64.2% were non-smokers, and the BRAF V600E mutation was more prevalent in men (52.8%). In addition, 96.2% of the patients had adenocarcinoma, and most (96.2%) received first-line therapy (23.5% anti-BRAF), with a progression-free survival (PFS) and overall survival (OS) of 10.0 [95% confidence interval (CI): 1.5–36.0 months] and 24.0 months [95% CI: 3.0–53.0 months], respectively. Twenty-three patients (43.4%) received second-line treatment (39.1% anti-BRAF), and PFS and OS were 5.0 [95% CI: 1.0–21.0 months] and 13.0 months [95% CI: 1.5–26.0 months], respectively. BRAF and MEK-targeted therapy (dabrafenib plus trametinib) produced longer PFS compared with that of chemotherapy with or without bevacizumab as a first-line (NA vs. 4.0 months, *P* = 0.025) or second-line therapy (6.0 vs. 4.6 months, *P* = 0.017). NSCLC patients harboring driver oncogene mutations such as BRAF V600E, EGFR, or ALK should be treated using targeted therapies. Concurrent TP53 mutations were the most common, affecting 11.3% (*n* = 6) of the patients, followed by EGFR 19 Del (*n* = 5). Patients with concurrent mutations had shorter PFS (9.0 vs. 10.0 months, *P* = 0.875) and OS (14.0 vs. 15.0 months, *P* = 0.555) than those without these mutations.

**Conclusion:**

These results suggest that combined BRAF- and MEK-targeted therapy is effective in BRAF V600E-mutated advanced NSCLC patients. Dabrafenib and trametinib re-challenge is also an option for patients with BRAF V600E-mutated NSCLC.

## Introduction

Recent non-small cell lung cancer (NSCLC) therapy research has concentrated on developing drugs targeting driver gene mutations, particularly for adenocarcinoma (1, 2). All patients with advanced adenocarcinoma undergo routine genetic testing for clinically targetable genomic alterations (3). Clinical targeted therapy is imperative for NSCLC patients with epidermal growth factor receptor (EGFR) mutation (4, 5). EGFR-targeting tyrosine kinase inhibitors (TKIs), such as gefitinib, erlotinib, and osimertinib, extend median progression-free survival (PFS) and overall survival (OS) in NSCLC patients with EGFR mutations (6, 7). Additionally, the anaplastic lymphoma kinase (ALK) inhibitor crizotinib has transformed treatment for NSCLC patients with ALK rearrangement (8–10). Currently, through the clinical application of comprehensive genome sequencing, many potential targetable genomic alterations, such as v-Raf murine sarcoma viral oncogene homolog B1 (BRAF), mesenchymal-epithelial transition (MET) exon 14 skipping mutations, HER2 (human epidermal growth factor receptor 2), and rearranged during transfection (RET) gene rearrangements, have been verified.

BRAF-encoded protein-RAF kinase is one of the main regulators of the MAPK/ERK pathway and activates downstream MEK through phosphorylation (11). The MAPK/ERK signaling pathway regulates cell growth, proliferation, differentiation, migration, and apoptosis (12–14). BRAF mutations occur in approximately 2–4% of NSCLC patients with adenocarcinoma (15, 16). In the BRAF oncogene, over 50% of mutations are associated with glutamate-valine substitution of the 600-position codon (V600E, Val600Glu) in the 15th exon of the kinase domain. This mutation results in a 500-fold increase in BRAF kinase activity compared with that of the wild-type (17). The BRAF V600E mutation is common in older patients (>60 years), patients with adenocarcinoma (18–20). Recently, the United States Food and Drug Administration approved BRAF (dabrafenib) and MEK inhibitor (trametinib) treatment for NSCLC patients with the BRAF V600E mutation. In a clinical trial of advanced NSCLC with BRAF V600E mutations, the combination of BRAF and MEK inhibitors (dabrafenib and trametinib) had a 64% overall response rate (ORR) in formerly untreated patients (21). In Europe, a real-world study assessed the efficacy of combined dabrafenib and trametinib in treating BRAF V600E mutated advanced NSCLC. Across the entire cohort, median PFS and OS were 17.5 months (95% confidence interval [CI] 7.1–23.0 months) and 25.5 months (95% CI 16.6–not reached), respectively (22). Another study reported that the OS in BRAF-targeted therapy was longer (56.5 months) than that of conventionally treated patients (27.2 months), further emphasizing the importance of targeting treatments for BRAF V600E mutated NSCLC (23).

In Chinese advanced NSCLC patients with the BRAF V600E mutation, the clinical efficacy of chemotherapy, immunotherapy, targeted therapy, and other conventional therapies has not been well explored. This is probably because the BRAF V600E mutation in NSCLC is rare, and there are no approved treatments targeting the mutation prior to the use of BRAF and MEK inhibitors. To address this issue, we conducted a retrospective study to assess the clinical characteristics and outcomes of advanced NSCLC patients with the BRAF V600E mutation.

## Materials and Methods

### Study Subjects and Data Collection

Clinical data from January 2015 to December 2021 were collected. The primary objective was to describe the clinical characteristics and determine the efficacy of dabrafenib and trametinib in advanced NSCLC patients with the BRAF V600E mutation. Fifty-three patients were recruited from multiple centers, including (I) the First Affiliated Hospital, College of Medicine, Zhejiang University; (II) Hunan Cancer Hospital, Affiliated Tumor Hospital of Xiangya Medical School of Central South University; and (III) Wenzhou Medical University. Patients at these centers who met the following inclusion criteria were enrolled in this study: (I) patients with a histological or cytological diagnosis of NSCLC; (II) patients with the BRAF V600E mutation detected by multiplex polymerase chain reaction (PCR) or next-generation sequencing (NGS); and (III) patients diagnosed with advanced NSCLC, including those with stage IV metastatic disease and with inoperable locally advanced stage IV disease. Using the response evaluation criteria in solid tumors (RECIST) version 1.1 (24), patients received an Eastern Cooperative Oncology Group (ECOG) performance status score to measure disease severity. After obtaining informed consent from the study patients, patient medical records were analyzed, and patient characteristics, including age, sex, smoking history, ECOG score, histology, staging, co-occurring mutations, and treatment history, were recorded. Disease staging was determined using the American Joint Council on Cancer (AJCC) Staging System, Version 8. This clinical study was approved by the Institutional Review Boards of the First Affiliated Hospital, College of Medicine, Zhejiang University, Hunan Cancer Hospital, and Wenzhou Medical University.

### Outcomes and Assessments

NSCLC patients with the BRAF V600E mutation received dabrafenib (150 mg BID) plus trametinib (2 mg QD) as first-line or follow-up therapy. Other therapies included platinum-based chemotherapy, immune checkpoint inhibitors (ICIs), EGFR-TKI, and ALK-TKI targeted therapy. The main objective of this clinical trial was to assess PFS and OS from initiation of treatment in patients with the BRAF V600E mutation. Using the Solid Tumor Version 1.1 (RECIST 1.1) criteria, PFS was defined as radiological or clinical progression (deterioration of clinical status, prophylactic systemic therapy) or death, while OS was defined from the start of therapy to death. Assessment was performed at each participating center without centralized imaging.

### Statistical Analysis

The Kaplan–Meier curve and log-rank statistics were used to analyze the survival of each group. All statistical and graphing analyses were performed using GraphPad Prism 8 (GraphPad Software, San Diego, CA, USA). A statistically significant difference was defined as a *P*-value < 0.05.

## Results

### Patient Characteristics

A total of 53 patients with BRAF V600E mutation advanced NSCLC met the inclusion criteria for this study (**Table 1**). The median age was 58 years (range: 40–75 years). The cohort included a higher proportion of men (28/53, 52.8%) than women (25/53, 47.2%), and 64.2% (34/53) of the patients were non-smokers. Most patients (51/53, 96.2%) had stage IV NSCLC, with only two patients (3.8%) diagnosed as stage IIIc. Most patients had an ECOG performance status of 0–1 (46/53, 86.8%). The location of metastases at the time of diagnosis differed, with bone (25/53, 47.2%) being the most common location, followed by pleura (16/53, 30.2%), lung (10/53, 18.9%), liver (7/53, 13.2%), and brain (6/53, 11.3%). There are several clinical methods for detecting the BRAF V600E mutation. In our study, PCR was the most common method (31/53, 58.5%), followed by NGS (22/53, 41.5%). The median follow-up time for each patient from diagnosis to the last follow-up was 16.8 months.

**Table 1:** Baseline Characteristics.

Total patients,n (%)	53	100%
**Gender**		
Male	28	52.8
Female	25	47.2
**Median age(years, range)**	58(40-75)	
**Smoking History**		
Non-smoker	34	64.2
Smoker	19	35.8
**ECOG status at initial diagnosis**		
0-1	46	86.8
≥2	7	13.2
**Histology**		
Adenocarcinoma	51	96.2
Squamous carcinoma	1	1.9
Large cell neuroendocrine carcinoma	1	1.9
**Stage**		
IIIc(Locally Advanced)	2	3.8
IV (Metastatic)	51	96.2
**Sites of distant metastases at first diagnosis**		
Bone	25	47.2
Pleura	16	30.2
Lung	10	18.9
Liver	7	13.2
Brian	6	11.3
Adrenal	5	9.4
Lymph nodes	4	7.5
**BRAF test method**		
Multiplex PCR	31	58.5
NGS	22	41.5
**PD-L1 expression**		
≥50%	3	5.7
1%-50%	7	13.2
Un-detect	43	81.1

ECOG, Eastern Cooperative Oncology Group; PCR, Polymerase chain reaction; NGS, Next generation sequencing.

### Systemic Therapy

**Figure 1:**
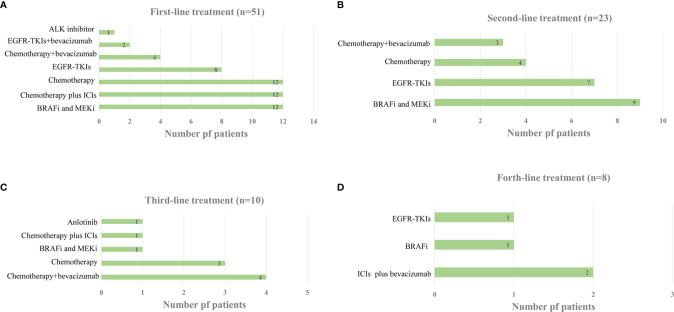
**(A)** Distribution of Systemic Treatment Regimens in the first-line Setting. **(B)** Distribution of Systemic Treatment Regimens in the second-line Setting. **(C)** Distribution of Systemic Treatment Regimens in the third-line Setting. **(D)** Distribution of Systemic Treatment Regimens in the forth-line Setting.

Of the 53 patients with advanced NSCLC who were scheduled to undergo first-line treatment, two refused treatment. At 31.4% (16/51), platinum-based dual chemotherapy with or without bevacizumab was the most common treatment regimen. Meanwhile, 23.5% (12/51) of the patients received the BRAF-MEK-directed therapy, dabrafenib-trametinib, as first-line therapy, while 23.5% (12/51) received platinum-based doublet chemotherapy combined with anti-programmed death-1 (PD-1) ICIs. For patients with EGFR/ALK combined with a BRAF V600E mutation, 15.7% (8/51) received EGFR-TKIs, 3.9% (2/51) received EGFR-TKIs plus bevacizumab, and 2.0% (1/51) received the EML4-ALK inhibitor crizotinib. The median duration of first-line treatment was 9.2 months (range: 1.5–36 months) (**Figure 1A**).

A total of 15 patients continued first-line therapy, whereas 11 gave up treatment after disease progression. Another two patients had to stop treatment because of severe renal insufficiency and infection after first-line therapy. Therefore, 23 patients received second-line therapy. BRAF and MEK inhibitors were administered to 39.1% (9/23) of second-line therapy patients, followed by EGFR-TKIs (7/23, 30.4%) and chemotherapy with or without bevacizumab (7/23, 30.4%) (**Figure 1B**). The median duration of second-line therapy was 6.2 months (range: 1.0–21.0 months). Additional data on subsequent therapy lines are presented in **Figures 1C, D**. Among the 51 patients who obtained at least one line of systemic therapy, 43.1% (21/51) received targeted treatment with BRAF/MEK inhibitors. Of these 21 patients, one received BRAF/MEK inhibitors as both first- and third-line therapies, with none receiving BRAF/MEK inhibitor therapy beyond fifth-line therapy.

### Progression-Free Survival Outcomes Analysis

**Figure 2:**
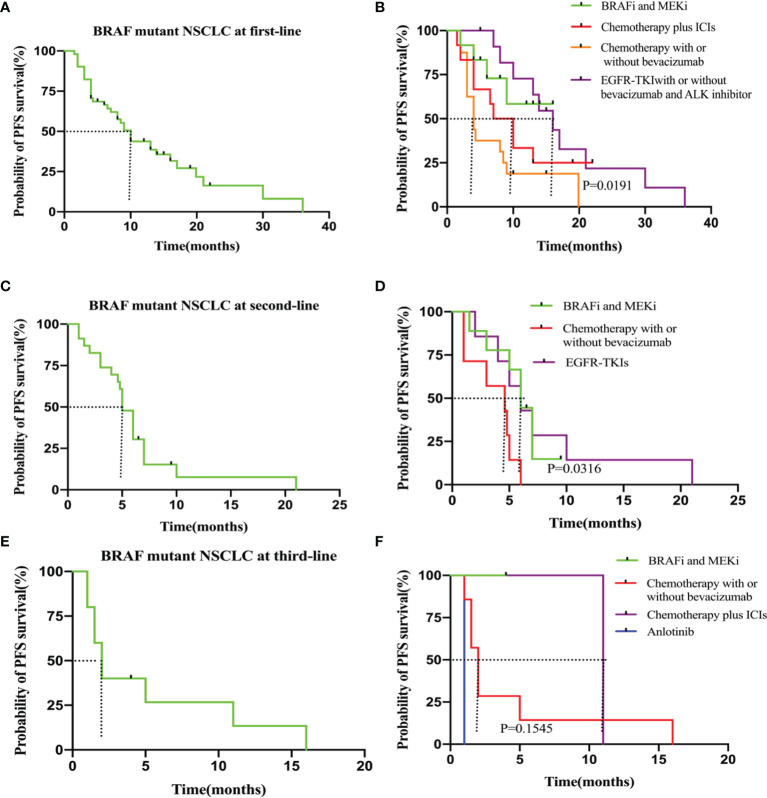
**(A)** PFS in the all patients at first-line. **(B)** PFS in the subgroup of patients who received BRAF and MEK inhibitor (n = 12), chemotherapy plus ICIs (n = 12), chemotherapy with or without bevacizumab(n=16) and EGFR-TKIs with or without bevacizumab/ALK inhibitor (n = 11). **(C)** PFS in the all patients at second-line. **(D)** PFS in the subgroup of patients who received BRAF and MEK inhitor (n = 9), chemotherapy with or without bevacizumab (n=7) and EGFR-TKIs (n=7). **(E)** PFS in the all patients at third-line. **(F)** PFS in the subgroup of patients who received chemotherapy with or without bevacizumab (n = 7), BRAF and MEK inhitor (n = 1), chemotherapy plus ICIs (n=2) and anlotinib (n=1).

In the 51 patients treated with systemic therapy, the median PFS from first-line therapy initiation was 10.0 months (95% CI, 1.5–36.0 months). The median first-line PFS was longer in the BRAF and MEK inhibitor cohorts compared with that in patients who received chemotherapy with or without bevacizumab (NA vs. 4.0 months, *P* = 0.025, hazard ratio [HR]: 0.33, 95% CI: 0.13–0.83). For patients who received EGFR-TKIs with or without bevacizumab/ALK inhibitor treatment, the median PFS was 16.0 months, with no significant difference compared with that of the BRAF and MEK inhibitor cohorts (16.0 months vs. NA, *P* = 0.710, HR: 1.20, 95% CI: 0.36–4.10). Regarding BRAF and MEK inhibitors, median PFS from first-line treatment did not significantly differ relative to that of patients who received chemotherapy plus ICIs (NA vs. 8.5 months, *P* = 0.211, HR: 0.49, 95% CI: 0.16–1.50) (**Figures 2A, B** and **Table 2**).

**Table 2:** Outcome on First-and later lines of therapy.

From start first-line therapy	No. Patients	PFS (months)	P value^*^ (95%CI)	OS (months)	P value^#^ (95%CI)
BRAFi and MEKi^&^	12	NA		NA	
Chemotherapy plus ICIs	12	8.5	0.211 (0.16-1.50)	14.0	0. 408 (0.18-2.00)
Chemotherapy with or without bevacizumab	16	4.0	0.025 (0.13-0.83)	14.0	0.528 (0.21-2.20)
EGFR-TKIs with or without bevacizumab/ALK inhibitor	11	16.0	0.710 (0.36-4.10)	33.0	0.039 (0.46-20.0)
**From start second-line therapy**					
BRAFi and MEKi	9	6.0		15.0	
Chemotherapy with or without bevacizumab	7	4.6	0.017 (0.10-1.10)	11.0	0.264 (0.15-2.00)
EGFR-TKIs	7	6.0	0.834 (0.33-4.00)	17.0	0.823 (0.34-3.70)
**From start third-line therapy**					
BRAFi and MEKi	1	NA		NA	
Chemotherapy with or without bevacizumab	7	2.0	0.276 (0.02-3.20)	13.0	0.578 (0.00-18.00)
Chemotherapy plus ICIs	1	11.0	/	12.0	/
Anlotinib	1	1.0	0.317 (0.00-6.80)	12.0	/
**From start forth-line therapy**					
BRAFi**^!^**	1	6.0		11.0	
ICIs plus bevacizumab	2	2.3	0.225 (0.03-3.20)	2.7	0.225 (0.03-3.20)
EGFR-TKIs	1	3.4	0.317 (0.01-8.20)	NA	/

*The P value of median PFS in BRAFi and/or MEKi cohort compare with another cohorts. #The P value of median OS in BRAFi and/or MEKi cohort compare with another cohorts. **^&^** BRAFi and MEKi included dabrafenib and combination dabrafenib.!BRAFi included vemurafenib.

NA, not arrived.

In the 23 patients who accepted second-line treatment, the median PFS was 5.0 months (95% CI: 1.0–21.0). The PFS was significantly longer for the nine patients who received BRAF and MEK inhibitor therapy than that for the seven patients who received chemotherapy with or without bevacizumab (6.0 vs. 4.6 months, *P* = 0.017, HR: 0.34; 95% CI: 0.10–1.10). The PFS was the same for BRAF/MEK inhibitor and EGFR-TKI therapies (6.0 vs. 6.0 months, *P* = 0.834, HR: 1.1, 95% CI: 0.33–4.00). Therefore, for patients without EGFR-sensitive mutations, we recommend BRAF and MEK inhibitor therapy as second-line therapy (**Figures 2C, D** and **Table 2**).

In the 10 patients who received third-line therapy, median PFS was shorter (2.0 months, 95% CI: 1.0–16.0) than for those who received first and second-line therapies. For the one patient who underwent BRAF and MEK inhibitor targeted therapy, median third-line PFS was not reached relative to the seven patients who received chemotherapy with or without bevacizumab (NA vs. 2.0 months, *P* = 0.276, HR: 0.23; 95% CI: 0.02–3.20). The Kaplan–Meier survival curves and outcomes for all PFS estimates are provided in **Figures 2E, F** and **Table 2**.

Ten patients tested positive for programmed death-ligand 1 (PD-L1) during the course of the disease, among whom, five received ICIs. Considering the various antibodies for PD-L1 and the sample size being too small in study patients, the relationships among PD-L1, BRAF mutation, and treatment efficacy were not analyzed. However, three patients had PD-L1 expression ≥50%, and their efficacy evaluation following first-line ICI therapy indicated a partial response (PR), with PFS of 13.0, 8.0, and 18.5 months. These results indicate that for patients with the BRAF V600E mutation, PD-L1 expression should be determined, and once the PD-L1 expression is ≥50%, ICIs may be an optimal choice for these patients.

### Overall Survival Outcomes Analysis

**Figure 3:**
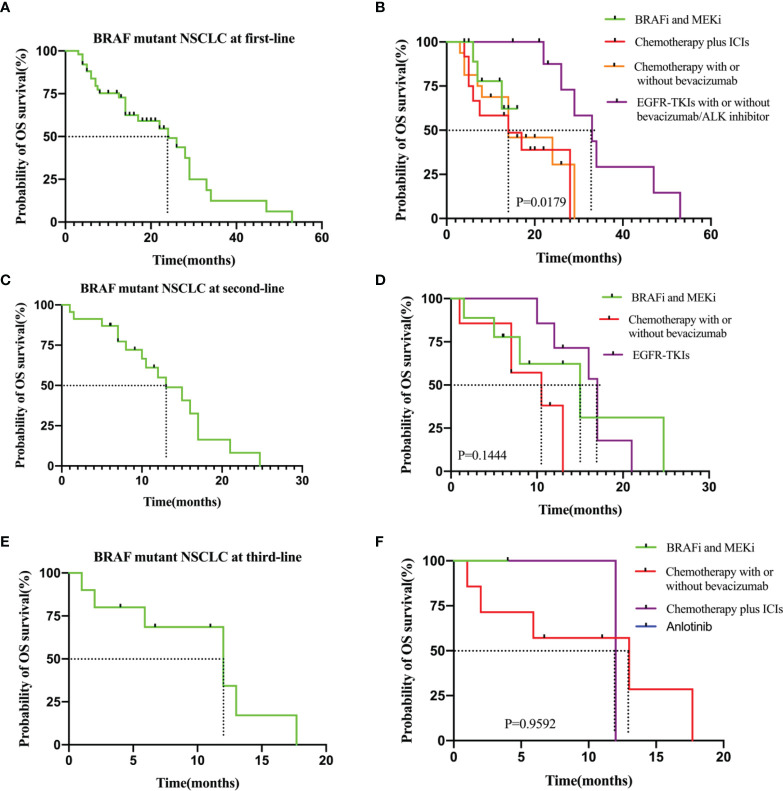
**(A)** OS in the all patients at first-line. **(B)** OS in the subgroup of patients who received BRAF and MEK inhitor (n = 12), chemotherapy plus ICIs (n = 12), chemotherapy with or without bevacizumab (n=16) and EGFR-TKIs with or without bevacizumab/ALK inhibitor (n = 11). **(C)** OS in the all patients at second-line. **(D)** OS in the subgroup of patients who received BRAF and MEK inhitor (n = 9), chemotherapy with or without bevacizumab (n=7) and EGFR-TKIs (n=7). **(E)** OS in the all patients at third-line.**(F)** OS in the subgroup of patients who received chemotherapy with or without bevacizumab (n = 7), BRAF and MEK inhitor (n = 1), chemotherapy plus ICIs (n=2) and anlotinib (n=1).

Among patients receiving first-line therapy, the median OS from the start of first-line therapy was 24.0 months (95% CI: 3.0–47.0). Median first-line OS was not reached for patients who received BRAF and MEK inhibitor targeted therapy, whereas it was 14.0 months for those who received chemotherapy plus ICIs (*P* = 0.408, HR: 0.56, 95% CI: 0.18–2.00). Median first-line OS did not significantly differ between the BRAF and MEK inhibitor cohort and the chemotherapy with or without bevacizumab cohort (NA vs. 14.0 months, *P* = 0.528, HR: 0.68, 95% CI: 0.21-2.20). For the patients treated with BRAF/MEK inhibitors and those treated with EGFR or ALK mutation targeted therapy, the median OS significantly differed (NA vs. 33.0 months, *P* = 0.039, HR: 3.0, 95% CI: 0.46–20.0) (**Figures 3A, B** and **Table 2**). The median OS for second- and third-line therapy was 13.0 (95% CI: 1.5–26.0) and 12.0 months (95% CI: 2.0–16.7), respectively. Patients receiving targeted treatment with BRAF and MEK inhibitors had a longer OS upon second-line therapy than that for patients receiving conventional therapy (except for EGFR-TKI therapy) (**Figures 3C, D** and **Table 2**). **Figures 3E, F** and **Table 2** show Kaplan–Meier survival outcomes for OS of third-line therapy patients.

### Concurrent Mutations

**Figure 4:**
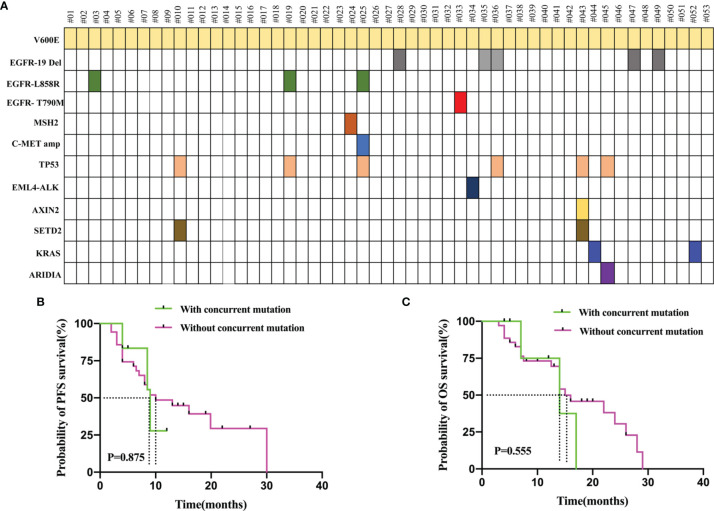
**(A)** Genomic Alterations Found in Each patient’s Tumor are Shown. 30.2% (16/53) of the *BRAF* mutation patients had other concurrent mutations. Concurrent TP53 mutations were the most common. **(B)** PFS in the patients who with concurrent mutation and without concurrent mutation. **(C)** OS in the patients who with concurrent mutation and without concurrent mutation. The concurrent mutation is excluded the sensitive mutations patients including EGFR and ALK positive.

PCR and NGS tests were performed in patients with advanced NSCLC to elucidate their baseline genetic mutation status. Results showed that 30.2% (16/53) of the BRAF mutation patients had other concurrent mutations, while the rest did not. Concurrent TP53 mutations were the most common, affecting 11.3% (6/53) of the patients. In addition, other co-mutations, including EGFR-19del (p.E746_A750del, *n* = 5), EGFR-L858R (leucine to arginine at position 858, *n* = 3), SETD2 mutation (*n* = 2), KRAS mutation (p.G12D, *n* = 1; p.G12C, *n* = 1), EGFR-T790M (substitutes methionine for threonine at amino acid position 790, *n* = 1), EML4-ALK rearrangement (*n* = 1), c-MET amplification (*n* = 1), MSH2 mutation (*n* = 1), AXIN2 mutation (*n* = 1), and ARIDIA mutation (*n* = 1) were also detected. The concurrent mutations observed are summarized in **Figure 4**. Furthermore, we determined the presence or absence of concurrent mutations associated with survival outcomes that excluded sensitive mutations in the 51 patients, including those that were EGFR- and ALK-positive (*n* = 10). Patients with concurrent mutations (*n* = 6) had shorter, albeit statistically non-significant, PFS (9.0 vs. 10.0 months, *P* = 0.875, **Figure 4B**) and OS (14.0 vs. 15.0 months, *P* = 0.555; **Figure 4C**) than those without (*n* = 35).

### Dabrafenib and Trametinib Re-Challenge

A 52-year-old female patient (case #10) came to our hospital, and a computed tomography (CT) scan detected a mass in the left lung, which was suspected to be cancer with multiple lymph node metastases to the left hilar mediastinum and both clavicles. Pleural effusion was also noted. Positron emission tomography/CT showed clavicular, mediastinal and peritoneal lymph node, adrenal gland, liver, right humeral, left knee, vertebral (T8,11, and 12), and bilateral ischial metastases, as well as multiple brain metastases. Thoracentesis fluid histology demonstrated adenocarcinoma cells. She was diagnosed and confirmed as having stage IV lung adenocarcinoma based on a bronchoscopic biopsy in August 2020. Immunohistochemical biopsy results revealed TTF-1, Napsin A, and CK7 positivity in the tumor cells. Genetic testing revealed a BRAF V600E mutation.

**Figure 5:**
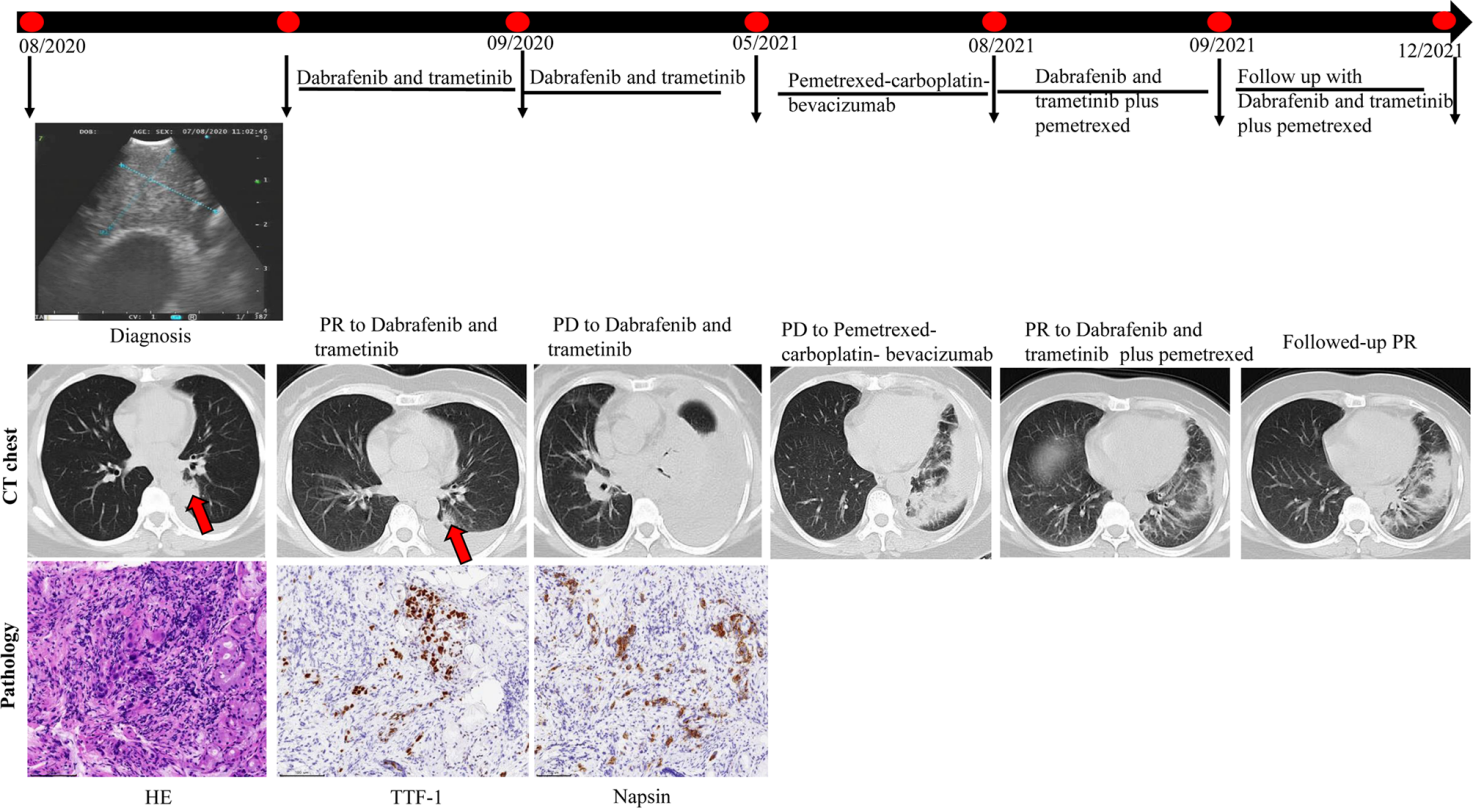
Computed tomography (CT) imaging reveals initial and re-challenge response to dabrafenib-trametinib combination therapy.

First-line therapy with dabrafenib-trametinib resulted in a PR in September 2020. Gamma knife treatment was also prescribed for the brain metastases. Significant symptom improvement and pleural effusion reduction were observed after treatment, and the patient was discharged with outpatient follow-up. Re-examination of brain magnetic resonance imaging 1 month post-gamma knife treatment showed reductions in the size of brain metastases. Nine months later (May 2021), an apparent increase in left pleural effusion was noted. A pemetrexed-carboplatin-bevacizumab combination was administered as second-line therapy. Three months later, the pleural effusion was still not controlled, and serum creatinine levels were also elevated. Therefore, puncture and drainage of pleural effusion were performed, in which adenocarcinoma cells were found by histology. BRAF V600E, TP53, and SETD2 mutations were also identified by targeted next-generation DNA sequencing of the pleural fluid samples. A dabrafenib-trametinib re-challenge plus pemetrexed was initiated. She achieved a PR in September 2021, which continued to date with no adverse effects noted (**Figure 5**). These findings indicate that dabrafenib-trametinib re-challenge is an alternative therapy for patients with the BRAF V600E mutation in NSCLC.

## Discussion

Little is known about the clinical features and treatment efficacy for patients with BRAF V600E mutated NSCLC, as the BRAF V600E mutation rarely occurs in NSCLC (25). Our cohort comprised patients with BRAF V600E-mutant advanced NSCLC, including 64.2% non-smokers with a slight male dominance (52.8%). Marchetti et al. proposed that patients with the BRAF-V600E mutation were significantly predominated by women or those who had never smoked (26). Ding et al. suggested that BRAF mutation in Chinese patients was more likely to occur in non-smokers (27). In terms of pathological features, BRAF-mutated NSCLC mostly comprises adenocarcinoma, and other histological types, including squamous cell carcinoma, have also been detected (28, 29). In our study, 96.2% (51/53) of the patients had the adenocarcinoma subtype. This clinical characteristic data is comparable with that of other studies. The 262 BRAF-mutated patients recorded by Barlesi et al. were characterized by an average age of 65.9 years, and 87% of the patients had adenocarcinoma (30).

In our cohort, 30.2% (16/53) of patients with the BRAF V600E mutation had other concurrent mutations. Concurrent TP53 mutations were the most common, affecting 11.3% (6/53) of the patients. The other concurrent mutations detected were EGFR-19del, EGFR-L858R, KRAS mutation, SETD2 mutation, EGFR-T790M, EML4-ALK rearrangement, and c-MET amplification. Co-occurrence rates in BRAF-mutated NSCLC are reported to be 14–16% (27, 31). KRAS Q61R mutation was examined in three patients who progressed before combined treatment with dabrafenib and trametinib (32). In other studies, KRAS mutation has been found in melanoma patients who progressed following combined BRAF and MEK inhibition therapy (33, 34). Another report revealed that KRAS mutation acts as a mechanism to resist BRAF and MEK inhibitors in NSCLC (35). In our study, a patient (case 44) with concurrent KRAS mutation still received treatment with dabrafenib-trametinib. Therefore, to determine whether KRAS co-mutation affects the response to BRAF-targeted therapy, further investigation is required to clarify the relationship between KRAS and BRAF. In addition, it has been reported that tumors containing TP53 co-mutations are related to worse clinical outcomes. Unfortunately, due to the limited number of clinical samples with BRAF V600E and TP53 co-mutations, the clinical implications of treatment for these patients were not studied.

Auliac previously reported that among the 46 advanced NSCLC patients with the BRAF V600E mutation registered in France between 2012 and 2014, those with the BRAF V600E mutation had 8.7 months of PFS with first-line therapy and 4.1 months with second-line therapy (36). In our study, patients with the BRAF V600E mutation had a PFS of 10.0 months (95% CI: 1.5–36.0) and OS of 24.0 months (95% CI: 3.0–53.0) with first-line therapy. The second-line therapy patients had a PFS of 5.0 months (95% CI: 1.0–21.0) and OS of 13.0 months (95% C: 1.5–26.0), consistent with Auliac (36). It is well known that NGS technologies have played an essential role in understanding the altered genetic pathways involved in human cancer. NGS can quantitate the proportion of reads for a given mutation, also known as mutant allele frequency (MAF), which represents the percentage of tumor cells that harbor a specific mutation in neoplastic tissue. One study investigated the value of BRAF V600E MAF variability within primary melanomas (MMp) and its potential prognostic implications. Results indicated that High-BRAFV600E MMp were all located on the trunk, had lower Breslow and mitotic indices, and were predominantly first nodal metastases. The high-BRAFV600E MMp patients had better prognostic features and first nodal metastasis (37). In the present study, we did not investigate whether the MAF of BRAF V600E potentially impacts the clinical outcome of NSCLC patients. In the future, we intend to pay attention to determine MAF in clinical practice for use as a prognostic indicator.

A retrospective multicenter study that included 40 advanced NSCLC patients with the BRAF V600E mutation treated with dabrafenib and trametinib was conducted. Among the nine patients receiving first-line therapy, median PFS and OS were 16.8 (95% CI: 6.1–23.2) and 21.8 months (95% CI: 1.0–not achieved), respectively. Median PFS and OS were 16.8 months (95% CI: 6.1–23.2) and 25.5 months (95% CI: 16.6 months–not met), respectively, in 31 patients receiving second-line therapy or above (22). In our study, 23.5% (12/51) and 39.1% (9/23) of the patients received BRAF/MEK-targeted therapy as first- and second-line therapies, respectively. The PFS and OS in patients who received dabrafenib-trametinib first-line treatment were not reached. The PFS and OS for second-line therapy patients who underwent dabrafenib-trametinib therapy were 6.0 and 15.0 months. The clinical role of dabrafenib and trametinib in advanced NSCLC patients with the BRAF V600E mutation was explored in an unselected real-world study. Among patients who did not receive prior treatment, PFS and OS were 10.8 (7.0–14.5) and 17.3 (12.3–40.2) months, respectively, whereas for formerly treated patients, these were 10.2 months (95% CI: 6.9–16.7) and 18.2 months (95% CI: 14.3–28.6), respectively (21, 38, 39). Our results were also consistent with the findings of other real-world studies. For instance, in a study where 31 patients received BRAF inhibitors, the median PFS for those on anti-BRAF treatment was 5.0 months, with an OS of 10.8 months (40).

In our study, the other first-line therapies included chemotherapy with/without bevacizumab, chemotherapy plus ICIs, and EGFR-TKIs with or without bevacizumab/ALK inhibitor. Other treatments used as second-line therapy were chemotherapy with or without bevacizumab and EGFR-TKIs. The PFS and OS in the first-line therapy patients who received chemotherapy with or without bevacizumab were 4.0 and 14.0 months, those for chemotherapy plus ICIs were 8.5 and 14.0 months, and for EGFR-TKIs with or without bevacizumab/ALK inhibitor 16.0 and 33.0 months, respectively. For second-line therapy, the PFS and OS for chemotherapy with or without bevacizumab were 4.6 and 11.0 months and those for EGFR-TKIs were 6.0 and 17.0 months, respectively. Zhuang conducted a study showing that first-line anti-BRAF-targeting treatment was superior to chemotherapy in 46 patients with advanced BRAF-V600E mutation (9.8 vs. 5.4 months, *P* = 0.149) (41). Similarly, in the present study, the PFS for first- and second-line BRAF/MEK-targeted therapy was also longer than that for chemotherapy with or without bevacizumab (first-line: NA vs. 4.0 months, *P* = 0.025; second-line: 6.0 vs. 4.6 months, *P* = 0.017), indicating that BRAF/MEK-targeted therapy is a viable choice for advanced NSCLC patients with the BRAF-V600E mutation. The PFS for first-, third-, and fourth-line therapies in the ICI-treated cohort was 8.5, 11.0, and 2.3 months, respectively. We intended to compare the PFS and OS between BRAF/MEK-targeted and ICI treatments; however, the PFS and OS in BRAF/MEK-targeted therapy were not reached. In fourth-line therapy, the PFS was longer for BRAF/MEK-targeted therapy than that for ICI treatment (6.0 vs. 3.4 months, *P* = 0.317). Another study by Gautschi reported that in the nine patients treated with ICIs, the median PFS was 3.0 months (40). Therefore, the effect of ICIs and therapeutic options for the BRAF V600E mutated population requires further exploration.

The retrospective study included patients diagnosed with NSCLC and tested for EGFR, ALK, ROS1, and BRAF mutations. Results showed that EGFR-TKI treatment was superior to chemotherapy in patients with BRAF V600E mutation concurrent with EGFR mutation (median PFS 10.8 vs. 5.2 months, *P* = 0.023) (41). Similarly, in the current study, the PFS and OS of patients treated with EGFR-TKIs with or without bevacizumab/ALK inhibitor were 16.0 and 33.0 months for first-line therapy, respectively. For second-line therapy, EGFR-TKI therapy was found to have a longer OS than that of BRAF/MEK-targeted therapy (17.0 vs. 15.0 months, *P* = 0.823). Therefore, in patients with the BRAF V600E-mutation and concurrent EGFR mutation, EGFR-TKIs may be the optimal treatment choice.

Current acquired resistance mechanisms to BRAF and MEK inhibitors in NSCLC patients are difficult to elucidate from molecular diagnosis. In our study, a patient with advanced NSCLC with BRAF V600E mutation (case 10) achieved a long-term PR to BRAF and MEK-targeted treatment re-challenge. The mechanism of action of response to re-challenge in BRAF and MEK-targeted therapy remains unclear. Reschke suggests that acquired resistance to BRAF and MEK inhibitors in metastatic melanoma might be reversible under “drug-free” conditions (42). Thus, cytotoxic chemotherapy creates a “drug-free” environment and may lead to re-challenge for some patients receiving BRAF and MEK-targeted therapy with positive outcomes. In another study, a patient with NSCLC with advanced BRAF V600E mutation was resistant to BRAF-targeted therapy in first-line therapy and therefore received chemotherapy as second-line therapy. After progression to chemotherapy, BRAF-targeted therapy was re-challenged, and the patient benefited (43).

The current study has some limitations. First, our study comprised a small sample of patients with BRAF V600E-positive NSCLC from three academic hospitals in China, and the results may not apply to other cancer centers. Second, the immunotherapy results for BRAF V600E-mutated NSCLC are limited and require further research. Therefore, due to a lack of consensus, final recommendations for immunotherapy or BRAF- and MEK-targeted therapy for patients with BRAF V600E mutation could not be reached. Third, we were unable to acquire tissue samples from patients with the BRAF V600E-mutation who were resistant to BRAF- and MEK-targeted therapy to further explore the mechanism of resistance to BRAF- and MEK-targeted therapy. In addition, we lacked an independent radiology review board to re-assess the outcomes at different medical centers. Therefore, multicenter prospective studies with a larger cohort of Chinese patients with the BRAF V600E mutation are needed.

## Conclusions

Our study uncovered differences in clinical characteristics and treatment efficacy in patients with BRAF V600E-mutated NSCLC in the Chinese population. Our data suggest that patients with NSCLC with carcinogenic alterations such as EGFR, ALK, and BRAF V600E may receive targeted treatment. PFS and OS were longer in patients receiving BRAF and MEK inhibitors in first- and second-line therapies than in those receiving chemotherapy. The value of ICI treatment in the BRAF V600E population requires further investigation. Patients with concurrent mutations had shorter PFS and OS than those without these mutations. Dabrafenib and trametinib re-challenge has potential as an alternative treatment for patients with NSCLC with advanced BRAF V600E mutation. Taken together, these findings suggest that BRAF- and MEK-targeting is a potential treatment option for patients with BRAF V600E mutated NSCLC.

## Data Availability Statement

The original contributions presented in the study are included in the article/supplementary material. Further inquiries can be directed to the corresponding authors.

## Ethics Statement

This clinical study was reviewed and approved by the Institutional Review Board of the First Affiliated Hospital, College of Medicine, Zhejiang University and Hunan Cancer Hospital and Wenzhou Medical University. The patients/participants provided their written informed consent to participate in this study. Written informed consent was obtained from the individual(s) for the publication of any potentially identifiable images or data included in this article.

## Author Contributions

JJQ, QS, and FSK collected and analyzed data; JJQ, QS, YPL, LL, JianyiZ, and JianyaZ wrote the manuscript. LL and JianyaZ were responsible for study conception and design and acquiring financial support. All authors have read and agreed to publish the current version of the manuscript.

## Funding

This work was supported by grants from the National Natural Science Foundation of China (No. 81802278), Medicine Health Technology Plan of Zhejiang Province, China (No. 2022KY150), and the Natural Science Foundation of Zhejiang Province (No. LGF22H160005).

## Conflict of Interest

The authors declare that the research was conducted in the absence of any commercial or financial relationships that could be construed as a potential conflict of interest.

## Publisher’s Note

All claims expressed in this article are solely those of the authors and do not necessarily represent those of their affiliated organizations, or those of the publisher, the editors and the reviewers. Any product that may be evaluated in this article, or claim that may be made by its manufacturer, is not guaranteed or endorsed by the publisher.
